# Vaccine hesitancy and knowledge regarding maternal immunization among reproductive age women in central Italy: a cross sectional study

**DOI:** 10.3389/fgwh.2023.1237064

**Published:** 2023-09-14

**Authors:** Viviana Moschese, Luigi De Angelis, Maria Vittoria Capogna, Simona Graziani, Francesco Baglivo, Adalgisa Pietropolli, Michele Miraglia Del Giudice, Caterina Rizzo, the Italian Society of Pediatric Allergology and Immunology (SIAIP) Vaccine Committee, V. Moschese

**Affiliations:** ^1^ Pediatric Immunopathology and Allergology Unit, Tor Vergata University Hospital, University of Rome Tor Vergata, Rome, Italy; ^2^ Department of Translational Research and New Technologies in Medicine and Surgery, University of Pisa, Pisa, Italy; ^3^ Department of Obstetrics and Ginecology, Casilino General Hospital, Rome, Italy; ^4^ Section of Gynecology and Obstetrics, Department of Surgical Sciences, Tor Vergata University Hospital, University of Rome Tor Vergata, Rome, Italy; ^5^ Department of Woman, Child and of General and Specialized Surgery, University of Campania “Luigi Vanvitelli”, Naples, Italy

**Keywords:** maternal immunization, vaccine, knowledge, hesitancy, pregnancy

## Abstract

**Background:**

Vaccination in pregnancy offers protection to the mother and the newborn. In Italy, influenza, pertussis, and COVID-19 vaccinations are recommended in pregnancy, but vaccination coverage is still far from the National Immunization Plan goals. We aimed to assess knowledge and attitude on maternal immunization in two groups of Italian women, in pregnancy and in reproductive age (non pregnant).

**Methods:**

A cross sectional study on Italian childbearing age women gathering information on their knowledge on maternal immunization and attitudes to receiving influenza and pertussis vaccines in pregnancy was carried out at the University of Rome Tor Vergata, between September 2019 and February 2020. Logistic and multinomial regressions were chosen as statistical tests for our analysis.

**Results:**

1,031 women participated in the survey by answering the questionnaire. Out of these, 553 (53.6%) women were pregnant, and 478 (46.4%) were in the reproductive age. 37% (204/553) of pregnant women and 41% (198/476) of non pregnant women are aware of the existence of an immunization plan for pregnant women in Italy. The group with age between 20 and 30, for both pregnant women and women in the reproductive age, has a better knowledge of vaccination in pregnancy. Working status is a variable associated with more awareness about vaccination during pregnancy only for pregnant women (OR = 2.34, *p* < 0.00001). Educational status, trimester of pregnancy and knowledge on the topic are associated with vaccine hesitancy in our multivariate analysis for pregnant women. In the reproductive age group women who had a previous pregnancy are more likely to be hesitant towards vaccination in pregnancy, on the other hand the one with a higher knowledge and educational status are more likely to get vaccinated.

**Conclusions:**

The study highlights the persistent vaccine hesitancy among Italian women of reproductive age and pregnant women. Despite healthcare providers being identified as a reliable source of information, their recommendations alone are insufficient to overcome vaccine hesitancy. Factors such as employment status, educational level, pregnancy trimester, and knowledge about vaccinations during pregnancy influence vaccine hesitancy. Tailored educational interventions and communication campaigns targeting these areas can help reduce vaccine hesitancy and promote maternal immunization.

## Introduction

Vaccination during pregnancy serves as a vital preventive measure to safeguard the health of both women and their newborns. The Italian Ministry of Health and international scientific authorities recommend the administration of the anti-influenza vaccine at any point during pregnancy, within the flu season (1), and the diphtheria-tetanus-acellular pertussis (DTaP) vaccine between the 27th and 36th weeks of gestation (2). Furthermore, since September 2021, in Italy Sars-CoV-2 mRNa vaccines are recommended to pregnant women in the second and third trimester in view of the growing evidence on the safety of these vaccines in both mother and newborn (3).

Pregnant women are particularly susceptible to severe influenza illness and related complications, which can lead to hospitalization and, in extreme cases, even death (4). Furthermore, influenza infections can negatively impact birth outcomes by causing prematurity, low birth weight, and perinatal mortality (5, 6). Given these potential risks, the anti-influenza vaccination is of paramount importance for protecting both mothers and their children from complications, while offering high tolerability and minimal adverse effects (7, 8).

Numerous studies have demonstrated that *Bordetella pertussis* commonly causes respiratory infections, which can result in severe complications in newborns (9). Maternal immunization has been proven not only effective in preventing neonatal pertussis (10) but also safe during pregnancy (11, 12).

Pregnancy also increases the likelihood of experiencing severe COVID-19 (13). Therefore, it is advised and advantageous for pregnant women to receive vaccination, as it also benefits newborns by reducing the chances of preterm birth and low 5-minute Apgar scores (14).

However, it is important to note that our questionnaire was conducted prior to the COVID-19 pandemic and did not consider vaccine knowledge and hesitancy specifically related to this issue.

In spite of the well-established benefits for both women and newborns (15, 16), vaccination coverage among pregnant women in Italy is alarmingly low (6, 17, 18), especially when compared to other countries (15, 19).

Indeed, vaccine coverage is variably reported according to the different settings and surveyed cohorts with Italian rates mostly ranging from 6% to 19% for flu, 5% to 61% for pertussis, and approximately of 20% for COVID-19 vaccines. These rates are tendentially lower than in other countries (20, 21). To mention some, amongst the Irish pregnant population the influenza and COVID-19 uptake rates were 62% and 25%, respectively; conversely, in UK, the Public Health England reported a seasonal influenza vaccine rate of 44% (22) whereas the most recent US estimates evaluated a rate of 61%, 57% and 70% for flu, pertussis and COVID-19 vaccines, respectively (23). Context-specific factors should be adequately addressed to better understand local barriers and devise efficient maternal immunization strategies that encourage successful vaccine uptake. By employing targeted educational interventions and communication campaigns, it may be possible to significantly improve maternal immunization rates (17). This, in turn, would contribute to the overall health and safety of mothers and their newborns, reducing the risks associated with vaccine-preventable diseases.

The primary objective of this study is to investigate the knowledge and vaccine hesitancy of Italian women regarding vaccinations during pregnancy in order to evaluate the underlying factors contributing to low vaccination coverage. Additionally, as a secondary objective, this study seeks to explore vaccine hesitancy in relation to pediatric vaccination among women who have children.

## Methods

### Study design

A cross-sectional study was carried out using a close-ended questionnaire in Italian distributed to childbearing age women accessing the Department of Obstetrics and Gynecology and the Pediatric Immunopathology and Allergology Unit of Tor Vergata University Hospital and Affiliated Centers of the University of Rome Tor Vergata, between September 2019 and February 2020. The study population included Italian women, pregnant at any gestational age and in reproductive age (non pregnant), who routinely attended the outpatient clinics for consultation in consecutive days for the aforementioned period. The participating women represented a heterogeneous cohort in terms of demographic, socioeconomic and cultural features representative of the general population.

### Questionnaire development

A close-ended questionnaire was developed specifically for this study to collect data on various aspects of reproductive health. The questionnaire was designed based on a thorough literature review and input from experts in the field (24, 25). It comprised multiple sections. The questionnaire consisted of 10 items including socio-demographic and pregnancy information, vaccine status of their child/children, reasons for vaccine refusal, knowledge on maternal immunization programs, source of information for vaccinations and attitudes to receiving vaccination in pregnancy (Supplementary Material 1).

### Data collection

Data collection was carried out in 6 months, during which the questionnaire was distributed on consecutive days to the childbearing age women who attended the outpatient clinics for a routine consultation. The questionnaire was available in paper format and provided by trained healthcare professionals to women sitting in the waiting room before consultation and after providing clear instructions on the purpose of the study and obtaining informed consent. Participation was voluntary with no payment or incentives to complete the questionnaire.

### Data analysis

Descriptive statistics, such as frequencies and percentages, were computed for categorical variables, while means and standard deviations were calculated for continuous variables. Logistic regression analyses were conducted to meet the objectives of the study. In order to meet our study objectives firstly, we conducted multiple univariate analyses where we examined each variable independently, without considering the effect of other variables, to evaluate its association with the outcomes of interest (knowledge about vaccination during pregnancy and vaccine hesitancy). We evaluated several factors such as working status, age class, pregnancy trimester (only for pregnant women), previous pregnancy, and education, and calculated their respective odds ratios (OR) and 95% confidence intervals (95% CI). First trimester and age >40 years were considered as reference groups in all the regression analysis. Subsequently, we conducted a multivariate logistic regression analysis to identify the variables that influenced knowledge about vaccination during pregnancy. We included as covariates in the multivariate mode only the variables that demonstrated significant association with the outcome in the univariate analysis. This allowed us to evaluate the independent impact of each variable on knowledge about vaccination during pregnancy, while controlling for the effect of other variables. Results were considered statistically significant in all our analysis with a *p*-value < 0.05. The study population was divided into pregnant and non pregnant women in reproductive age and results were compared between these two groups.

All data were collected into an EXCEL database (Microsoft, Redmond, Washington—United States) and the analysis was performed using R software.

### Ethical considerations

The study was conducted according to the Declaration of Helsinki and was approved by the Ethics Committee of Tor Vergata University Hospital, Rome, Italy (53/18).

## Results

Of the 1,100 women approached, 1,031 agreed to partecipate in the survey by answering the questionnaire and giving informed consent, with a response rate of 94%. Out of these 1,031 women, 553 (53.6%) were pregnant, and 478 (46.4%) were in the reproductive age. The characteristics of the study population are summarized in Table 1. Among pregnant women, 157 (28.4%) were in the first trimester, 107 (19.3%) in the second, and 289 (52.3%) in the third trimester.

**Table 1:** Summary of the characteristics of the study population, including age class, employment status, education level and having children, for pregnant and reproductive age women (non pregnant) who participated in the study.

Pregnant women population									
Age class	*n*°	Employed	Has children	I Trimester	II Trimester	III Trimester	Middle school	High school	University
<20	17	0 (0%)	2 (11.8%)	5 (29.4%)	3 (17.6%)	9 (52.9%)	1 (5.9%)	16 (94.1%)	0 (0%)
20–30	154	79 (51.3%)	68 (44.1%)	34 (22.1%)	30 (19.5%)	90 (58.4%)	11 (7.1%)	118 (76.6%)	25 (16.2%)
30–40	316	231 (73.1%)	188 (59.4%)	96 (30.4%)	61 (19.3%)	159 (50.3%)	24 (7.6%)	168 (53.2%)	124 (39.2%)
>40	66	55 (83.3%)	41 (62.1%)	22 (33.3%)	13 (19.7%)	31 (47.0%)	2 (3.0%)	34 (51.5%)	30 (45.4%)
**TOTAL**	**553**	**365** **(66.0%)**	**299** **(54.1%)**	**157** **(28.4%)**	**107** **(19.3%)**	**289** **(52.3%)**	**38** **(6.9%)**	**336** **(60.7%)**	**179** **(32.4%)**
Reproductive age women population (non pregnant)									
Age class	*n*°	Employed	Has children	Middle school	High school	University			
<20	29	4 (13.8%)	1 (3.4%)	7 (24.1%)	22 (75.9%)	0 (0%)			
20–30	144	58 (40.3%)	38 (26.4%)	7 (4.9%)	98 (68.0%)	39 (27.1%)			
30–40	158	126 (79.7%)	126 (79.7%)	5 (3.2%)	81 (51.3%)	72 (45.6%)			
>40	147	119 (80.9%)	129 (87.7%)	7 (4.8%)	102 (69.4%)	38 (25.9%)			
**TOTAL**	**478**	**307** **(64.2%)**	**294** **(61.5%)**	**26** **(5.4%)**	**303** **(63.4%)**	**149** **(31.2%)**			

We also include questions about the information sources deemed reliable for maternal immunization (Item 10). The results presented in Table 2 demonstrate the preferences of pregnant and non-pregnant women in selecting their trusted sources for information related to maternal immunization. Both groups showed a similar pattern, with physicians, midwives, and nurses being the most preferred source, followed by media outlets, internet, and pharmacists.

**Table 2:** Summary of reliable information sources for maternal immunization (item 10) among pregnant and reproductive age women (non pregnant), including the number and percentage of respondents who selected each source.

Reliable information source for maternal immunization	Pregnant women	Reproductive age women
Physicians, midwives, and nurses	510 (92.2%)	455 (95.2%)
Media (magazines, newspapers, and TV)	52 (9.4%)	31 (6.8%)
Internet (social networks and blogs)	44 (7.9%)	29 (6.1%)
Pharmacists	28 (5.1%)	30 (6.3%)
Non-medical friends and family members	7 (1.3%)	6 (1.2%)
Pharmaceutical companies	9 (1.6%)	4 (0.8%)

### Factors associated with knowledge about vaccination during pregnancy: logistic regression analysis

Results show that 37% (204/553) of pregnant women and 41% (198/476) of non pregnant women are aware of the existence of specific national immunization recommendations for pregnant women in Italy.

For pregnant women, working status (OR = 2.18, 95% CI = 1.49–3.23, *p*-value < 0.0001), age class between 20 and 30 (OR = 2.11, 95% CI = 1.14–4.03, *p*-value < 0.05), third trimester of pregnancy (OR = 1.92, 95% CI = 1.31–3.06, *p*-value < 0.01) were statistically associated with knowledge of recommended vaccination during pregnancy. Other variables (previous pregnancy, educational level) were analyzed with univariate logistic regression, but were not found to be associated with the outcome (Supplementary Table S1).

Similarly, for women in the reproductive age group, we conducted univariate analysis for every socio-demographic factor: only age class between 20 and 30 (OR = 2.15, 95% CI 1.35–3.45, *p*-value < 0.001) and previous pregnancy (OR = 0.56, 95% CI 0.39–0.82, *p*-value < 0.001) showed to be correlated with the outcome. Among these women, working status and education did not demonstrate a significant association with knowledge about vaccination during pregnancy (Supplementary Table S1).

In the multivariate analysis, we adjusted for the effects of other variables to evaluate the independent association of each variable with the primary outcome. For pregnant women, the factors that remained significantly associated with the outcome in the multivariate analysis were working status (OR = 2.34, 95% CI 1.55–3.58, *p* < 0.00001), third trimester of pregnancy (OR = 1.95, 95% CI 1.27–3.03, *p* < 0.001) and age class between 20 and 30 (OR = 2.64, 95% CI 1.38–5.18, *p*-value < 0.001). For women in the reproductive age group, only the age group between 20 and 30 was significantly associated with the outcome in the multivariate analysis (OR = 1.76, 95% CI 1.02–3.07, *p*-value < 0.05) (Supplementary Table S2).

Overall, our findings suggest that the group with age between 20 and 30, for both pregnant women and women in the reproductive age, has a better knowledge of vaccination in pregnancy.

Working status is a variable associated with more awareness about vaccination during pregnancy only for pregnant women.

### Vaccine hesitancy of mothers regarding pediatric vaccination

We also investigated vaccine hesitancy regarding pediatric vaccination among the 603 women in our study who had children (Table 3). Of these, 580 (96.2%) reported to have regularly vaccinated their children. However, 23 (3.8%) mothers reported that they decided to not vaccinate their children. The most common reasons for vaccine hesitancy were fear and concern about vaccine side effects (10/23; 43.5%), preference for natural immunity (7/23; 30.4%), and fear of a possible causal link between vaccines and autism (5/23; 21.7%). A small proportion of women reported fearing vaccine additive side effects (3/23; 13%), vaccine efficacy (3/23; 13%), over-stimulation of the immune system (2/23; 8.7%), sickness after vaccine administration (2/23; 8.7%), and costs (1/23; 4.3%).

**Table 3:** Distribution of reasons for vaccine hesitancy among a subset of mothers (23 out of 603) who chose not to vaccinate their children.

Reported reason for pediatric vaccine hesitancy	Number of hesitant mothers (tot = 23)	Percentage (%)
Fear of vaccine side effects	10	43.5
Preference for natural immunity	7	30.4
Fear of link between vaccines and autism	5	21.7
Fear of vaccine additive side effects	3	13.0
Doubts about vaccine efficacy	3	13.0
Fear of over-stimulation of the immune system	2	8.7
Fear of sickness after vaccine administration	2	8.7
Concern about costs	1	4.3

Each reason is accompanied by the number of mothers who reported it and the corresponding percentage out of the 23 hesitant mothers. Note that some mothers reported more than one reason, so the percentages add up to more than 100%.

### Vaccine hesitancy of women regarding maternal immunization in pregnancy

In Table 4 are reported the answers related to the willingness to get vaccinated during pregnancy in case of a health professional advice, provided by women in the 2 different populations (pregnant and reproductive age women). Among the 98 women answering “No”, the most common motivations were “fear and concern about the possible complications of vaccination” 42/98 (42.8%), and “concern about the possible adverse effects of vaccine excipients” 12/98 (12.2%).

**Table 4:** Summary of the responses of pregnant and reproductive age women (non pregnant) to item 9 (If a health professional advises you to get vaccinated during pregnancy, would you do it?), including the number and percentage of participants who responded “No”, “I don't know”, or “Yes”) for each age group.

Age class	Pregnant women				Reproductive age women			
	Total	No	I don't know	Yes	Total	No	I don't know	Yes
<20	17	5 (29%)	10 (59%)	2 (12%)	29	0 (0%)	22 (76%)	7 (24%)
20–30	154	18 (12%)	82 (53%)	54 (35%)	144	12 (8%)	56 (39%)	76 (53%)
30–40	316	34 (11%)	170 (54%)	112 (35%)	158	18 (11%)	86 (54%)	54 (34%)
>40	66	4 (6%)	39 (59%)	23 (35%)	147	7 (5%)	76 (52%)	64 (44%)
Total	553	61 (11%)	301 (54%)	191 (35%)	478	37 (8%)	240 (50%)	201 (42%)

Another objective of this study was to evaluate factors associated with vaccine hesitancy towards vaccination during pregnancy. Results of the univariate multinomial logistic regressions analysis are reported in Table 5 (more details in Supplementary Tables S3, S4), for vaccine hesitancy in both populations.

**Table 5:** Univariate analysis of factors associated with vaccine hesitancy in pregnant women and reproductive age women (non pregnant) (based on item 9 responses), including age class, education level, working status, pregnancy trimester, gynecologist communication, and knowledge about vaccinations.

Factor	Odds ratio (OR)	Significance	Hesitancy association
Univariate analysis item 9 in pregnant women			
Age (<20 years)	4.88	*p* < 0.00001	More hesitant
Working status (Not working)	0.50	*p* < 0.00001	More hesitant
Working status (Working)	2.52	*p* < 0.00001	Less hesitant
Education (University level)	0.44	*p* < 0.00001	Less hesitant
Education (Middle school)	0.53	*p* < 0.001	More hesitant
Pregnancy trimester (1st)	0.56	*p* < 0.05	More hesitant
Gynecologist communication	2.75	*p* < 0.0001	Less hesitant
Knowledge about vaccinations	6.55	*p* < 0.00001	Less hesitant
Univariate analysis item 9 in reproductive age women			
Age (<20 years)	0.38	*p* < 0.05	More hesitant
Education (University level)	0.47	*p* < 0.05	Less hesitant
Education (High school)	0.60	*p* < 0.05	Less hesitant
Previous pregnancy	0.59	*p* < 0.01	More hesitant
Gynecologist communication	3.10	*p* < 0.001	Less hesitant
Good gynecologist communication	2.16	*p* < 0.01	Less hesitant
Knowledge about vaccinations	4.43	*p* < 0.0001	Less hesitant

Only statistically significant results are reported, with odds ratios (OR), significance levels, and hesitancy associations presented accordingly.

For pregnant women, the results of the multivariate analysis showed that those who were working were more likely to be less hesitant towards vaccination during pregnancy (OR = 1.83, 95% CI 1.10–3.05, *p* < 0.05), while those with a middle school education level were more likely to be hesitant (OR = 1.90, 95% CI 1.02–3.54, *p* < 0.05). Conversely, university level education was associated with less hesitancy (OR = 0.53, 95% CI 0.30–0.92, *p*-value < 0.05).

Pregnant women in their third trimester were also more likely to be hesitant towards vaccination during pregnancy with an odds ratio of 0.55 (95% CI 0.39–0.76, *p* < 0.0001). While those with greater knowledge about vaccinations during pregnancy were significantly less hesitant (OR = 4.50, 95% CI 2.82–7.18, *p*-value < 0.000001).

For women in the reproductive age, the multivariate analysis revealed that those with a university-level education were less hesitant towards vaccination during pregnancy (OR = 0.40, 95% CI 0.21–0.84, *p*-value < 0.05). Women who had a previous pregnancy were more likely to be hesitant (OR = 0.56, 95% CI 0.32–0.98, *p*-value < 0.05), while those with greater knowledge about vaccinations during pregnancy were 3.88 times less hesitant (OR = 3.88, 95% CI 2.37–6.35, *p*-value < 0.000001).

## Discussion

In the last decades the importance of immunization during pregnancy has been widely demonstrated in those countries where specific vaccine programs have been implemented (26–28). Immunization in pregnancy is beneficial both for mothers and children (29–31). However, scientific evidence does not match vaccine adhesion, especially when involving children and pregnant women. In the present study, vaccine knowledge, awareness and behavior among Italian childbearing age women and pregnant women have been investigated.

Several studies have previously reported that approximately 25% of women are aware of the existence of maternal immunization programs (32), 39% of Italian women in our sample reported to be aware of the existence of vaccination programs for pregnant women.

Previous studies analyzed the reasons for vaccine hesitancy in pregnancy showing that 29% of women considered the vaccine harmful for the development of the fetus and almost 18% believed that vaccination did not protect infants against pertussis during the first months of life (17). In our study fear and concern of adverse events were especially related to vaccine excipients. Interestingly, despite the large diffusion of internet use among younger women, we observed that over 90% of women in both groups identified healthcare providers (HCPs) as a reliable information source for maternal immunization. Doctors, midwives and nurses are considered the main and most reliable source of information, as previously reported in other studies (33, 34). Trust in information provided by HCPs does not seem to be enough to overcome vaccine hesitancy, as only 38% (392/1,031) of women would vaccinate in pregnancy if advised by HCPs.

Unlike other studies (35), we observed that employed pregnant women are best informed. We hypothesize that occupational doctors might play a role in this, as they have the opportunity to provide information on vaccinations to pregnant women at work. It's important to highlight that in Italy at least one consultation with occupational doctors during pregnancy is necessary to obtain a certificate of leave from work, other consultations are optional.

Women in the third trimester of pregnancy are more likely to be aware of the existence of recommended vaccines in pregnancy, as they probably had more contacts with the gynecologist and, more importantly, the third trimester (between 27th and 36th week of pregnancy) is the period in which pertussis vaccination should be performed in Italy (28).

The age group between 20 and 30 years have reported a better knowledge of immunization programs for pregnant women, this could be explained by a border access to information (e.g., internet and social media) in this age group, this factor might increase their knowledge independently from the information provided by HCPs. Another hypothesis to explain the higher knowledge observed in this age group, is that women between 20 and 30 years are the target for a HPV vaccination campaign in Italy, with an active call for all the 25 years old women who are invited to do the HPV-DNA test and the HPV vaccine (if not vaccinated) (36). This could raise awareness also on other existing vaccinations in those young women.

Women in reproductive age who had a previous pregnancy in the univariate analysis results to have a lower knowledge of vaccination programs, but this result is not confirmed in the multivariate analysis, as this effect was presumably influenced by the women's age, being the only factor that remained statistically significant in the multivariate analysis.

Talking about the hesitancy of mothers towards pediatric vaccines, among the women who had at least one child at the time of the interview, 23 (3.8%) declared that their children had not been vaccinated. This result is comparable with the rate of parent's vaccine refusals reported in the literature (35, 37, 38), and the main reasons behind this choice are reported to be fear and concern about vaccine side effects, in particular a well-known anti-vax position such as the possible causal link between vaccines and autism is frequently reported. One limitation of relying on self-reported vaccine refusal is the potential for mothers to provide inaccurate information regarding the vaccination status of their children. This discrepancy may arise due to the fear of facing consequences for not adhering to mandatory vaccinations (the hexavalent DTaP-HepB-IPV-Hib and the quadrivalent MMR-V) imposed by the national immunization plan.

Further analysis on this topic could be possible only by having access to the regional or national vaccination registries. The main focus of our study is to better understand the phenomenon of vaccine hesitancy towards vaccination in pregnancy, especially because vaccine coverage in this population in Italy is alarmingly low (39, 40).

Only 35% of pregnant women in each age group, excluding the one under 20 years, would get vaccinated in pregnancy if advised. Our multivariate analysis suggests that employed women, with a higher educational status and a greater knowledge about the topic, are the one more likely to get vaccinated in pregnancy. Working and educational status could be both intended as social determinants, known to generally influence the attitude towards vaccination (41).

Young age (<20 years) is associated in the univariate analysis with a higher vaccine hesitancy in both pregnant and not-pregnant women, even if the level of statistical significance is low due to the small number of women under 20 years represented in our sample. Pregnant women in their third trimester, despite being more aware of the existence of vaccination programs were more likely to be hesitant. This controversial result might be explained by the fact that coming closer to the delivery, women could not perceive the need of vaccination for themselves and not be aware of the role of vaccines in pregnancy to protect their newborns.

Women of reproductive age who have had a previous pregnancy are more likely to exhibit hesitancy towards vaccination during pregnancy. This hesitancy may be influenced by their previous pregnancy experience, particularly if it was uncomplicated and both the mother and newborn were healthy. A positive previous pregnancy experience could potentially create a dangerous sense of reassurance or confidence, leading to increased hesitation towards vaccination.

In both groups, effective communication by gynecologists was found to be associated with lower vaccine hesitancy. This effect can only be seen in the univariate analysis because in the multivariate it's overshadowed by the variable on vaccine's knowledge. As one would imagine, knowledge about vaccination in pregnancy is a good predictor of a positive attitude towards these vaccines, this result highlights the crucial role of educational interventions and communication campaigns. The relevant communicative role of HCPs has been frequently raised (20, 42–44), since the lack of correct and detailed information is considered one of the main barriers to immunization during pregnancy. Vaccine refusal is strictly related to the fear of vaccine safety due to the lack of information by HCPs (32). A higher attitude is observed when women identify vaccines as an essential tool to protect their newborns, as reported by an online survey carried out in the United Kingdom (45).

Our results suggest that education level, employment status, pregnancy trimester, and knowledge about vaccinations during pregnancy are all crucial factors that influence vaccine hesitancy. Direct targeting of some of these areas is extremely challenging; knowledge on this topic is the most addressable factor. Educational interventions delivered using communication media and social media for the general population could be valuable in increasing knowledge on vaccination in pregnancy, targeting populations with lower knowledge based on their educational and employment status. It's also crucial to empower healthcare providers (especially general practitioners, pediatricians and gynecologists) that should properly inform pregnant women on the benefits of vaccinations in pregnancy.

Our results are similar to previous Italian studies (17, 21, 46), this shows how knowledge of vaccination programs for pregnant Italian women is almost unchanged over the years. Conversely, in other countries a more favorable attitude towards maternal vaccination has been observed. Particularly, in Canada and in Nicaragua women favorable to vaccines were 89% and 95%, respectively (47, 48).

A significant limitation of our study arises from the distribution of the questionnaire exclusively through a hospital setting. While this approach provided access to a specific sample group, it also inadvertently excluded certain segments of the population with less access to healthcare services. Pregnant women who do not frequently engage with hospitals or who receive prenatal care through alternative channels may have been underrepresented in our study. This limitation hampers the ability to draw conclusions that apply to these specific groups and highlights the need for caution when generalizing the findings to the entire pregnant population.

Additionally, the availability of the sole Italian language version of the questionnaire may have introduced a potential bias in participant selection. Since the questionnaire was not offered in languages other than Italian, individuals not fluent in Italian may have been excluded from the study.

The relevant communicative role of HCPs has been frequently raised (20, 42–44), since the lack of correct and detailed information is considered one of the main barriers to immunization during pregnancy.

As a result, it is essential for scientific societies to focus their efforts not only on emphasizing the significance of vaccination as a vital tool for protecting against infectious diseases but also on regulating and supporting the involvement of pregnant women in vaccine research.

By adopting this strategy, awareness about immunization among women of childbearing age would improve, leading to higher vaccine acceptance and uptake. Additionally, establishing dedicated maternal vaccine units in hospitals could streamline the process of providing vaccine information and administering vaccines.

The findings of this study call for increased research efforts, improved healthcare provider training, and targeted educational interventions. These actions are crucial to protect both maternal and fetal health promoting immunization during pregnancy.

## Data Availability

The raw data supporting the conclusions of this article will be made available by the authors, without undue reservation.

## Italian Society of Pediatric Allergology and Immunology (SIAIP) Vaccine Committee

Coordinator: V. Moschese, Pediatric Immunopathology and Allergology Unit, Policlinico Tor Vergata, University of Rome Tor Vergata, Rome, Italy. Centers: A. Beni, Department of Clinical and Experimental Medicine, University of Pisa, Pisa, Italy; G. Costagliola, Section of Clinical and Laboratory Immunology, Division of Pediatrics, Department of Clinical and Experimental Medicine, University of Pisa, Pisa, Italy; B. Martire, UOC of Pediatrics and Neonatology, “Monsignor A.R. Dimiccoli” Hospital, Barletta, Italy; M.F. Mastrototaro, UOC of Pediatrics and Neonatology, “Monsignor A.R. Dimiccoli” Hospital, Barletta, Italy; D. Montin, Division of Pediatric Immunology and Rheumatology, Department of Public Health and Pediatrics, “Regina Margherita” Children Hospital, University of Turin, Turin, Italy; G. Ottaviano, Department of Pediatrics, Fondazione IRCCS San Gerardo Dei Tintori, Monza, Italy; C. Rizzo, Department of Translational Research and New Technologies in Medicine and Surgery, University of Pisa, Pisa, Italy; V. Santilli Academic Department of Pediatrics (DPUO), Research Unit of Clinical Immunology and Vaccinology, IRCCS Bambino Gesù Children's Hospital, Rome, Italy; M. Sgrulletti, Pediatric Immunopathology and Allergology Unit, Policlinico Tor Vergata, University of Rome Tor Vergata, Rome, Italy and PhD Program in Immunology, Molecular Medicine and Applied Biotechnology, University of Rome Tor Vergata, Rome, Italy; G.L. Marseglia (past President), Department of Pediatrics, IRCCS Foundation, Policlinico San Matteo, University of Pavia, Pavia, Italy; M. Miraglia Del Giudice (President), Department of Woman, Child and of General and Specialized Surgery, University of Campania “Luigi Vanvitelli”, Naples, Italy.
