# Supplementary material for: Vaccine hesitancy and knowledge regarding maternal immunization among reproductive age women in central Italy: a cross sectional study
Source: Front Glob Womens Health. 2023 Sep 14;4:1237064. doi: 10.3389/fgwh.2023.1237064 (PMC10539584; doi:10.3389/fgwh.2023.1237064)
Supplement: Supplementary file 1 [file Datasheet1.pdf]

Survey Questionnaire

1. What stage of pregnancy are you in?

a) First Trimester

b) Second Trimester

c) Third Trimester

d) In childbearing age but not pregnant

2. What is your age?

a) < 20 years

b) 20 – 30 years

c) 30 – 40 years

d) > 40 years

3. What is your highest educational qualification?

a) Middle school

b) High school

c) University

4. Are you employed?

a) Yes

b) No

5. How many children do you have?

a) 0

b) 1

c) 2

d) 3

e) > 3

6. If you already have children, have they been regularly vaccinated?

a) Yes

b) No

6.bis If you answered NO to item 6:

What are the reasons that led you to this behavior (multiple answers possible):

a) Fear and concern about vaccine side effects

b) Fear of the possible causal link between vaccines and autism

c) Fear of vaccine additive side effects

d) Fear of immune system over-stimulation

e) Fear of getting sick due to the vaccine

f) Pain and stress due to the vaccine

g) Doubts about the need and effectiveness of vaccination

h) Preference for "natural" immunity

i) High Cost

j) Other

7. Has your gynecologist or midwife told you about vaccinations during pregnancy?

a) Yes, in a thorough way

b) Yes, but not in a thorough way

c) No

8. Are you aware that pregnant women can receive some vaccinations?

a) Yes

b) No

8.bis If you answered YES to item 8:

From who did you get this information?

a) Healthcare professional

b) People not involved in the healthcare field (relatives, friends)

c) Other information sources (internet, newspapers)

9. If a health professional advises you to get vaccinated during pregnancy, would you do it?

a) Yes

b) No

c) I don't know

9. bis If you answered NO to item 9:

What are the reasons to stay unvaccinated (multiple answer possible)?

a) Fear and concern about vaccine side effects

b) Fear of vaccine additive side effects

c) Fear of immune system over-stimulation

d) Fear of getting sick due to the vaccine

e) Pain and stress due to the vaccine

f) Doubts about the need and effectiveness of vaccination

g) Preference for "natural" immunity

h) High Costs

i) Other

10.Which of the following sources do you most frequently rely on to find information about maternal immunization?

a) Media (Magazines, newspapers and TV)

b) Physicians, midwives, and nurses

c) Pharmaceutical companies

d) Pharmacists

e) Non-medical friends and family members

f) Internet (social networks and blogs)
