# Supplementary material for: Vaccine hesitancy and knowledge regarding maternal immunization among reproductive age women in central Italy: a cross sectional study
Source: Front Glob Womens Health. 2023 Sep 14;4:1237064. doi: 10.3389/fgwh.2023.1237064 (PMC10539584; doi:10.3389/fgwh.2023.1237064)
Supplement: Supplementary file 2 [file Datasheet2.pdf]

SUPPLEMENTARY MATERIALS

Table S1 Univariate Analysis Results for Knowledge of Pregnancy Vaccination Across Different Subgroups

This table presents the results of univariate logistic regression analysis investigating the factors associated with knowledge of pregnancy vaccination among two distinct subsets of women: those currently pregnant, and those of reproductive age. The predictors considered include employment status, age category, trimester of pregnancy (for pregnant women), number of children (for reproductive age women), and education level (for reproductive age women). First trimester and age >40 were considered as reference groups. For each predictor, we report the estimated logistic regression coefficient (Estimate), standard error of the estimate (Std. Error), z-value, and associated p-value. It's important to note that odds ratios and confidence intervals were calculated only for the results with a p-value less than 0.05, which are considered statistically significant.

Subset of Population	Predictor	Estimate	Std. Error	z-value	p-value	Odds Ratio	95% CI
**Pregnant Women**	**Work (Yes)**	**0.7780**	**0.1979**	**3.931**	**8.47e-05**	**2.177033**	**1.485038, 3.230006**
Pregnant Women	Age (< 20)	0.1349	0.4707	0.286	0.7747	NA	NA
**Pregnant Women**	**Age (20 -30)**	**0.7460**	**0.3205**	**2.328**	**0.019936**	**2.1085271**	**1.14033191, 4.0274759**
Pregnant Women	Age (30 -40)	-0.2121	0.3225	-0.657	0.5110	NA	NA
Pregnant Women	Parity (yes)	0.1159	0.1772	0.654	0.513	NA	NA
Pregnant Women	Pregnancy trimester (second)	-0.2343	0.2168	-1.081	0.2796	NA	NA
**Pregnant Women**	**Pregnancy trimester (third)**	**0.6893**	**0.2148**	**3.209**	**0.00133**	**1.922389**	**1.3147388, 3.056280**
Reproductive Age Women	Work (Yes)	-0.3422	0.9130	-1.773	0.0762	NA	NA
Reproductive Age Women	Age (< 20)	-0.1274	0.4262	-0.299	0.76503	NA	NA
**Reproductive Age Women**	**Age (20 -30)**	**0.7658**	**0.2393**	**3.200**	**0.00137**	**2.1506494**	**1.3494158, 3.4516915**
Reproductive Age Women	Age (30 -40)	-0.1977	0.2402	-0.823	0.41046	NA	NA
**Reproductive Age Women**	**Parity (yes)**	**-0.5730**	**0.1910**	**-3.0**	**0.0027**	**0.5638298**	**0.3872074, 0.8192701**
Reproductive Age Women	Education (Middle school)	-0.74210	0.47199	-1.572	0.116	NA	NA
Reproductive Age Women	Education (High school)	-0.08344	0.20218	-0.413	0.6820	NA	NA

TABLE S2: Multivariate Analysis Results for Knowledge of Pregnancy Vaccination Across Different Subgroups

This table presents the results of multivariate logistic regression analysis investigating the factors associated with knowledge of pregnancy vaccination among two distinct subsets of women: those currently pregnant, and those of reproductive age. The predictors considered include only the one that were found to be significantly associated with the outcome in the univariate analysis. First trimester and age >40 were considered as reference groups. For each predictor, we report the estimated logistic regression coefficient (Estimate), standard error of the estimate (Std. Error), z-value, and associated p-value. It's important to note that odds ratios and confidence intervals were calculated only for the results with a p-value less than 0.05, which are considered statistically significant.

Subset of Population	Predictor	Estimate	Std. Error	z-value	p-value	Odds Ratio	95% CI
**Pregnant Women**	**Work (Yes)**	**0.8493**	**0.2136**	**3.976**	**7.00e-05**	**2.3379314**	**1.54828905, 3.5813870**
Pregnant Women	Age (< 20)	-1.1112	1.0871	-1.022	0.30671	NA	NA
**Pregnant Women**	**Age (20 -30)**	**0.9691**	**0.3354**	**2.889**	**0.00386**	**2.6356785**	**1.38444692, 5.1817186**
Pregnant Women	Age (30 -40)	0.5225	0.3059	1.708	0.08758	NA	NA
Pregnant Women	Pregnancy trimester (second)	0.3344	0.2780	1.203	0.22912	NA	NA
**Pregnant Women**	**Pregnancy trimester (third)**	**0.6686**	**0.2207**	**3.030**	**0.00245**	**1.9514905**	**1.27259231, 3.0270575**
Reproductive Age Women	Age (< 20)	-0.4017	0.4728	-0.850	0.3955	NA	NA
**Reproductive Age Women**	**Age (20 -30)**	**0.5679**	**0.2808**	**2.023**	**0.0431**	**1.7645910**	**1.0190580, 3.070280**
Reproductive Age Women	Age (30 - 40)	-0.2254	0.2416	-0.933	0.3510	NA	NA
Reproductive Age Women	Parity (yes)	-0.3261	0.2430	-1.342	0.1795	NA	NA

Table S3: Univariate Analysis Results of Vaccination Hesitancy in the subset of Pregnant Women

This table presents the results of the univariate analysis examining the association between various predictor variables and vaccination hesitancy among the subset of pregnant women. The analysis was performed using logistic regression models. The table includes the p-values and Odd Ratio (OR) for each predictor variable. The predictor variables examined in the analysis include age groups, work status, education level, number of children, communication with gynecologists and knowledge of pregnancy vaccination. First trimester and age >40 were considered as reference groups

Variable	Odds Ratio (Neutral as baseline)	p-value (Neutral as baseline)
**Age**		
**No (< 20)**	**4.875186**	**< 0.00001**
**Yes (< 20)**	**0.339093**	**< 0.00001**
**No (20-30)**	**2.14044**	**0.0368**
Yes (20–30)	1.11665	0.1861
No (30-40)	1.95019	0.1941
Yes (30-40)	1.11713	0.7269
**Work**		
**No**	**0.49792**	**< 0.00001**
**Yes**	**2.52453**	**< 0.00001**
**Education**		
**No (Middle school)**	**2.0283**	**< 0.00001**
**Yes (Middle school)**	**0.5276**	**0.0003**
**No (High school)**	**0.4302**	**0.0116**
Yes (High school)	0.8975	0.0733
**No (University)**	**0.43951**	**< 0.00001**
Yes (University)	1.3339	0.4802
**Gynecologist Communication**		
**No (Communication)**	**3.6852**	**< 0.00001**
**Yes (Communication)**	**10.7486**	**< 0.00001**
No (Good communication)	0.7976	0.6540
**Yes (Good communication)**	**2.7506**	**0.0001**
**Knowledge of Pregnancy Vaccination**		
No	1.5502	0.1628
**Yes**	**6.5480**	**< 0.00001**

Table S4: Univariate Analysis Results of Vaccination Hesitancy in the subset of Reproductive Age Women

This table presents the results of the univariate analysis examining the association between vaccine hesitancy and various factors among reproductive age women. The analysis includes stratification by age group, education level, and gynecologist communication. First trimester and age >40 were considered as reference groups. Odds ratios (OR) and p-values are provided for each comparison, with the neutral attitude as the baseline category. The odds ratios indicate the strength and direction of the association between each factor and vaccine hesitancy, while the p-values indicate the statistical significance of the associations.

Variable	Odds Ratio (Neutral as baseline)	p-value (Neutral as baseline)
**Age**		
No (< 20)	1.714e-05	0.9484
**Yes (< 20)**	**0.379**	**0.0368**
No (20-30)	2.327	0.0959
Yes (20–30)	1.612	0.0509
No (30-40)	2.273	0.0823
Yes (30-40)	0.746	0.2267
**Work**		
No	0.59934	0.1516
Yes	0.89326	0.5732
**Education**		
No (Middle school)	0.46987	0.9002
Yes (Middle school)	1.04116	0.9736
**No (High school)**	**0.6024**	**0.0385**
Yes (High school)	0.8621	0.3048
**No (University)**	**0.4699**	**0.0165**
Yes (University)	1.0412	0.8091
**Parity**		
No	0.80614	0.5557
**Yes**	**0.59315**	**0.0081**
**Gynecologist Communication**		
No (Communication)	0.96850	0.9609
**Yes (Communication)**	**3.0995**	**0.0003**
No (Good communication)	0.5055	0.3704
**Yes (Good communication)**	**2.1565**	**0.0098**
**Knowledge of Pregnancy Vaccination**		
**No**	**0.31298**	**0.0342**
**Yes**	**4.43142**	**< 0.0001**
